# Water restriction increases oxidation of endogenous amino acids in house sparrows (*Passer domesticus*)

**DOI:** 10.1242/jeb.246483

**Published:** 2024-03-15

**Authors:** Elizabeth J. Rogers, Alexander R. Gerson

**Affiliations:** ^1^ Organismic and Evolutionary Biology Program, University of Massachusetts Amherst, Amherst, MA 01003, USA; ^2^ Department of Biology, University of Massachusetts Amherst, Amherst, MA 01003, USA

**Keywords:** Water balance, Metabolism, Body composition, Stable isotopes, Birds

## Abstract

Animals can cope with dehydration in a myriad of ways, both behaviorally and physiologically. The oxidation of protein produces more metabolic water per kilojoule than that of fat or carbohydrate, and it is well established that birds increase protein catabolism in response to high rates of water loss. However, the fate of amino acids mobilized in response to water restriction has not been explicitly determined. While protein catabolism releases bound water, we hypothesized that water-restricted birds would also oxidize the resulting amino acids, producing additional water as a product of oxidative phosphorylation. To test this, we fed captive house sparrows (*Passer domesticus*) ^13^C-labeled leucine for 9 weeks to label endogenous proteins. We conducted weekly trials during which we measured the physiological response to water restriction as changes in lean mass, fat mass, metabolism and the enrichment of ^13^C in exhaled CO_2_ (δ^13^C_breath_). If water-restricted birds catabolized proteins and oxidized the resulting amino acids, we expected to simultaneously observe greater lean mass loss and elevated δ^13^C_breath_ relative to control birds. We found that water-restricted birds catabolized more lean tissue and also had enriched δ^13^C_breath_ in response to water restriction, supporting our hypothesis. δ^13^C_breath_, however, varied with metabolic rate and the length of the water restriction period, suggesting that birds may spare protein when water balance can be achieved using other physiological strategies.

## Teaser

**Summary:** Birds oxidize more endogenous amino acids under acute water stress, supporting the hypothesis that dehydrated birds catabolize protein to offset water losses by synthesizing endogenous water.

## INTRODUCTION

Water is a critical requirement for life and animals have evolved a diversity of behavioral and physiological adaptations to ensure that water loss and water gain remain balanced. Inputs to animal body water pools arise from two major sources: ingestion or absorption of water, and endogenous synthesis of metabolic water (Whiteman et al., 2019). In response to dehydration, animals may increase water input or reduce rates of water loss such as by concentrating urine, suppressing activity, or selecting more osmotically favorable microclimates (Schmidt-Nielsen, 1997). While animals can often respond to thirst and dehydration by ingesting more water, or by increasing cutaneous permeability, they may be reliant on endogenous water sources in environments where free water is scarce or during long fasts. Metabolic water production is dependent on an animal's metabolic rate and the relative mixture of oxidative fuels, which vary in both energy and water content. Fuel mixture composition depends on factors such as diet (Denommé et al., 2021; Gannes, 2001; Guglielmo et al., 2017; Voigt et al., 2012), exercise type or intensity (McCue et al., 2015; Suarez et al., 2009; Weber, 2011; Welch et al., 2011), and nutritional status (Cherel et al., 1988; McCue, 2012; McCue and Pollock, 2013).

In a fasting state, endogenous proteins are usually spared until other metabolic fuels have been depleted (Cherel et al., 1988; Jenni-Eiermann and Jenni, 2012). Birds are exceptionally good at storing and utilizing large amounts of fat to fuel energetically demanding activities (Blem, 1976; Ramenofsky, 1990; Guglielmo, 2010), yet for reasons not well understood, birds break down substantial amounts of lean tissue during long-distance flights despite having sufficient fat stores (Battley et al., 2000; Bauchinger and Biebach, 2001; Gerson and Guglielmo, 2013; Groom et al., 2019; Piersma, 1998). Protein catabolism during long flights may serve several non-exclusive functions, including but not limited to gluconeogenesis, replacement of intermediates for the citric acid cycle, endogenous protein turnover or water production (Bauchinger and McWilliams, 2010; Jenni and Jenni-Eiermann, 1998; Jenni-Eiermann and Jenni, 1991). Relative to fat and carbohydrate, protein yields approximately 5 times more bound and metabolic water per kilojoule when oxidized (Jenni and Jenni-Eiermann, 1998). Supporting the hypothesis that birds catabolize protein for water, rates of lean mass loss have been shown to increase under conditions of water restriction and high evaporative water loss (Gerson and Guglielmo, 2011a,b). Though overall rates of protein catabolism increase roughly linearly with metabolic rate (Jenni and Jenni-Eiermann, 1998), which may in part be explained by increased requirements for anaplerotic replacement of metabolic intermediates to support high rates of fatty acid oxidation, fasting birds burn roughly the same proportion of protein during flight and at rest (Jenni-Eiermann and Jenni, 2012). Differences in lean mass loss caused by water stress occur independently of metabolic rate in both exercising and inactive fasting birds (Gerson et al., 2020; Groom et al., 2019), indicating that increased rates of protein catabolism are not exclusively driven by anaplerosis or constitutive tissue degradation. While the current body of evidence suggests that protein catabolism plays a critical role in water balance and osmoregulation in birds, the fate of amino acids mobilized in response to water stress is unknown.

Dehydrated birds typically maintain consistent body water content and plasma volume (Dawson et al., 1979; Pinshow and Porter, 1992). One hypothesis is that birds increase the proportion of protein in their fuel mixture when osmotically stressed to offset water losses by increasing water input from metabolism. In this case, amino acids may be transported to the liver and oxidized, ultimately producing metabolic water via oxidative phosphorylation, which would supplement the bound water that is released by the breakdown of proteins. In addition to elevated rates of lean mass loss, dehydrated house sparrows exhibit increased plasma uric acid, adding support to the hypothesis that mobilized amino acids are oxidized for water (Gerson and Guglielmo, 2011a). Increased concentrations of plasma uric acid may also reflect mechanisms to concentrate urine in response to dehydration such as reduced glomerular filtration rate (GFR) or increased tubular water reabsorption. However, evidence suggests that birds do not alter GFR during flight or in response to respiratory water loss (Gerson and Guglielmo, 2013). Alternatively, protein catabolism may be important for establishing concentration gradients that facilitate the movement of water into and out of cells. Dehydrated birds maintain plasma volume through the movement of intracellular water to blood vessels down the osmotic gradient, a process driven by increased plasma osmolality (Carmi et al., 1994). Maintaining plasma volume may be a critical adaptation for birds that regularly experience high heat loads or sustain high metabolic rates for long periods, as plasma volume influences the ability of birds to efficiently dissipate heat and maintain a sufficient oxygen supply to tissues (Carmi et al., 1993). To reduce subsequent cellular dehydration, birds may break down proteins to increase intracellular concentrations of amino acids and peptides to bring plasma and intracellular osmolality towards equilibrium (Haussinger, 1996).

To sort among these hypotheses, we tested whether lean mass loss during dehydration in birds is driven by increased protein oxidation. Towards this end, we employed ^13^C breath testing to identify differences in the ^13^C/^12^C isotope ratio (δ^13^C) of exhaled CO_2_ in hydrated and dehydrated house sparrows (*Passer domesticus*). ^13^C breath testing can be used to trace oxidation of macronutrients based on known differences in δ^13^C between lipids and non-lipids due to isotopic fractionation (reviewed in McCue and Welch, 2016). Lipids are naturally depleted in ^13^C compared with non-lipids (i.e. carbohydrates and proteins), resulting in a lower δ^13^C by 5–15‰ (Welch et al., 2016). This difference can be magnified by feeding animals artificially enhanced tracers, such as ^13^C-labeled molecules, to make relatively small changes in metabolism easier to identify.

In the present study, we fed house sparrows a diet including ^13^C-labeled leucine, an essential amino acid, to enrich their tissues for ^13^C. As protein does not have a specialized storage form, animals rapidly oxidize surplus amino acids consumed in the diet (Bender, 2012; Wu, 2009). Of these dietary amino acids, non-essential amino acids, which can be resynthesized from other macromolecules, are oxidized at a much higher rate in house sparrows (McCue et al., 2010). Though some exogenous leucine may be oxidized immediately upon absorption, there is little ^13^C discrimination between essential amino acids in consumer tissues and those in the diet, suggesting that essential amino acids that are not immediately oxidized are incorporated directly into new tissues (Newsome et al., 2014). Tissues with high protein turnover are expected to have higher rates of isotopic incorporation (Carleton and Martínez del Rio, 2005), resulting in closer similarity with the isotopic composition of the animal's diet. In house sparrows, ^13^C resides in splanchnic tissues for 5–10 days and in pectoralis muscle for 20–30 days (Carleton et al., 2008). A study employing similar techniques to study the effect of water restriction on protein oxidation in mice found that dehydrated mice had greater cumulative oxidation of endogenously incorporated ^13^C-leucine after 72 h of fasting (McCue et al., 2017). Differences between hydrated and water-deprived mice were lower than predicted, however, suggesting that the protein-for-water strategy may be particularly beneficial in uricotelic animals such as birds and reptiles that can excrete nitrogenous waste as uric acid with minimum water loss.

To determine whether amino acid oxidation is elevated in house sparrows in response to dehydration, we conducted two separate experiments using established water restriction protocols to stimulate protein catabolism in birds on both control and ^13^C-labeled diets. We predicted that if birds under acute water stress elevate rates of endogenous amino acid oxidation, we would observe simultaneous reductions in lean mass and enrichment of ^13^C in the breath in water-restricted birds. Alternatively, if water-stressed birds catabolize proteins, but do not immediately oxidize mobilized amino acids, we expected reductions in lean mass but no enrichment of ^13^C in the breath relative to control birds. The two experiments differed in two primary ways: (1) the duration of the water restriction period prior to collecting metabolic measurements and (2) the length of time birds were in respirometry. In the first experiment, we conducted 12 h overnight respirometry to capture high-resolution shifts in fuel use during the middle stages of dehydration. As birds in respirometry are food restricted by design, we expected metabolic shifts to occur due to effects from both prolonged fasting and water availability. To minimize the effect of food restriction and maximize the difference in water restriction duration between treatment groups, we conducted a second experiment during which metabolic data were only collected during the final 2 h of the water restriction period.

## MATERIALS AND METHODS

### Animal care

We caught house sparrows, *Passer domesticus* (Linnaeus 1758) (*n*=10 males and *n*=4 females, sexed by plumage) using mist nets and potter traps in March 2021 near the University of Massachusetts, Amherst campus (Amherst, MA, USA). We moved birds to the animal care facility within 1 h of capture where we weighed, individually color banded and placed birds in groups of 2–3 in cages with *ad libitum* water and a diet of ground Mazuri Small Bird Diet mixed with white millet seed. We kept birds on a 12 h:12 h light:dark cycle for the duration of the experiment; lights turned on at 07:00 h and off at 19:00 h. After a 2 week acclimation period, we randomly assigned birds to two diet groups: we fed the unlabeled group (*n*=7) ground Mazuri Small Bird Diet and the labeled group (*n*=7) ground Mazuri Small Bird Diet with 0.2 g kg^−1 13^C-labeled leucine. To prepare the labeled diet, we dissolved ^13^C-1-l-leucine in water and mixed it completely with the bird's food, which we then dried in an incubator at 60°C overnight. We initiated the experiment 1 week after switching birds to the experimental diets; we kept them on these diets for the duration of the experiment. The University of Massachusetts, Amherst Institutional Animal Care and Use Committee approved all procedures (protocol #2825).

### Experimental design

#### Experiment 1

**Fig. 1.**
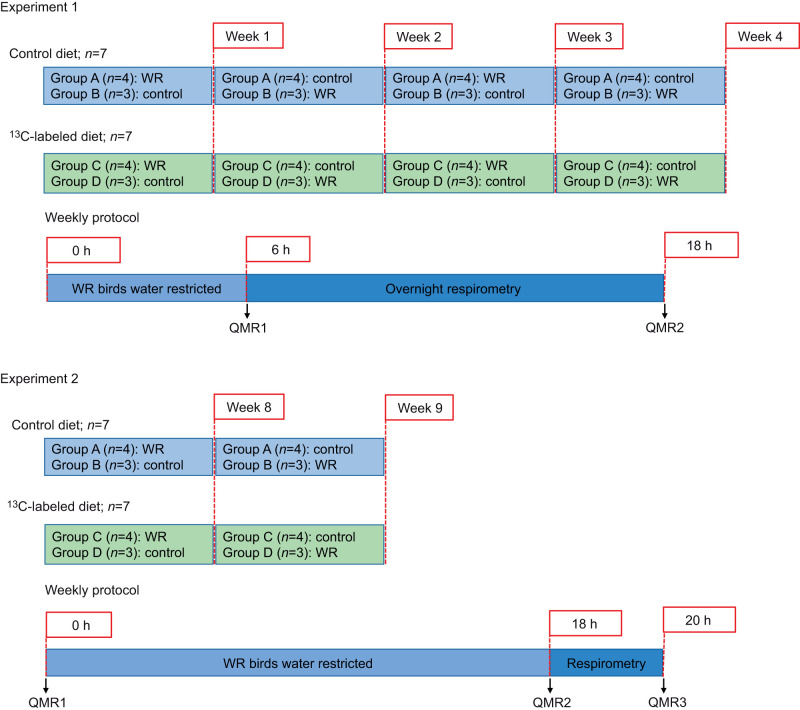
**Protocols for experiment 1 and experiment 2.** We initiated experiment 1 after switching birds to the experimental diets [unlabeled (control) and ^13^C-labeled]; experiment 2 started after the birds had been on the experimental diets for 7 weeks. We switched birds from control to water restricted (WR) treatments each week. The two experiments differed in the total length of time birds were water restricted, the duration and timing of the respirometry trials, and the frequency of body composition scans using quantitative magnetic resonance (QMR).

We randomly assigned birds in each diet group to two experimental treatments (*n*=3 and *n*=4), balanced by sex, which for each trial were either water restricted (WR) or control. For each trial, we performed the same experimental procedure on the diet groups separately and on consecutive days (Fig. 1). We repeated the procedure on both diet groups 4 times, with 1 week between trials. Individual birds were switched from WR or control each trial, so that each bird was only water restricted a maximum of once every 2 weeks. One day before each trial, we switched birds on the labeled diet to the control diet to minimize the ^13^C signal in the breath from unassimilated leucine. On each experimental day, we removed water from the cages of the WR birds at 12:00 h. We removed food from the cages of both WR and control birds 4 h later and assumed the birds to be post-absorptive 2 h after that. We then weighed and scanned all birds in triplicate using quantitative magnetic resonance (QMR; EchoMRI-B, Echo Medical Systems, Houston, TX, USA) for body composition analysis. We placed birds in individual, sealed 1 liter respirometry chambers in an environmental chamber (KB055, Darwin Chambers, St Louis, MO, USA) maintained at 24°C and 37% relative humidity (RH) for 12 h to measure resting metabolic rate (RMR) and the stable carbon isotope ratio of exhaled CO_2_ (δ^13^C_breath_), during which neither WR nor control birds had access to food or water. At the conclusion of the respirometry trial, we weighed and scanned birds in triplicate a second time using QMR.

#### Experiment 2

To minimize the time birds were in respirometry, and to maximize the difference in water restriction times for WR and control birds, we conducted a modified experimental procedure on both diet groups two additional times (Fig. 1). The experiment proceeded as above except for the following modifications. At 17:00 h on each experimental day, we weighed and scanned WR and control birds in triplicate using QMR. We then returned birds to their cages and removed water from the cages of the WR birds only. After 16 h, we removed food from the cages of both WR and control birds. We assumed the birds to be post-absorptive 2 h later, at which point we weighed and scanned birds in triplicate using QMR. We then placed birds in individual, sealed 1 liter chambers in an environmental chamber (KB055, Darwin Chambers) maintained at 30°C and 40% RH for 2 h to measure RMR and δ^13^C_breath_. At the conclusion of the respirometry trial, we weighed and scanned birds with QMR in triplicate a third time.

### Respirometry

We used standard flow-through respirometry techniques to measure RMR simultaneously with evaporative water loss (EWL) and δ^13^C_breath_. Each respirometry chamber received dry, CO_2_-free incurrent air at a constant flow of 400–600 ml min^−1^ (measured after the chambers with an SS-4 Sub-Sampler Flow Meter, Sable Systems International, Las Vegas, NV, USA). We measured excurrent air CO_2_ and H_2_O using a LI-840A CO_2_/H_2_O gas analyzer (LI-COR, Lincoln, NE, USA) and O_2_ using a FC-10 oxygen analyzer (Sable Systems International). We used a G2131-I isotopic carbon analyzer (Picarro, Santa Clara, CA, USA) to measure δ^13^C_breath_. Using multiplexing, we simultaneously collected data using Expedata (Sable Systems International) on 7 birds in 5 min intervals, with a 5 min baseline every 35 min. We lag corrected fractional concentrations of O_2_, CO_2_ and H_2_O and calculated *V̇*_O_2__ (ml min^−1^), *V̇*_CO_2__ (ml min^−1^) and *V̇*_H_2_O_ (mg min^−1^) from the mean of the final 2 min of each 5 min sampling interval using standard equations for push respirometry, and to account for water dilution effects (Lighton, 2018). For experiment 1 only, we calculated the total volume of O_2_ consumed and total water loss during respirometry (hours 6–18 of the water restriction period) by finding the area under the curve for the *V̇*_O_2_ _and *V̇*_H_2_O_ traces.

### Statistical analysis

We performed all statistics in R (v.4.2.0; http://www.R-project.org/) and analyzed data from experiments 1 and 2 separately. We investigated differences in body composition and metabolism between WR and control birds using linear mixed models (function ‘lmer’ from package lme4) with individual ID as a random effect for repeated measures. We selected all models using backward stepwise selection with a cutoff of α=0.05 for fixed effects. To reduce the effect of noise from the QMR machine on body composition measurements, we selected and averaged the two closest replicate measurements for fat (g) and lean mass (g) for each individual and time point. Initial models for body composition (total, lean and fat mass) included time point, treatment, diet and a time point by treatment interaction as fixed effects. For body composition change models, fixed effects included treatment, diet and initial body mass.

We calculated RMR by taking the average of the two lowest *V̇*_O_2__ measurements. We analyzed the *V̇*_CO_2__, *V̇*_H_2_O_ (hereafter evaporative water loss, EWL), respiratory quotient (RQ; calculated as *V̇*_CO_2__/*V̇*_O_2__) and δ^13^C_breath_ values taken during the sampling intervals corresponding to RMR. Because of an error measuring excurrent CO_2_ concentration in chambers 3 and 4 during weeks 1–3 of the first experiment, we removed those measurements from our analysis. For RMR, our starting model included initial fat, initial lean mass and treatment. The models for EWL and RQ additionally included RMR as a covariate. For δ^13^C_breath_, we included *V̇*_CO_2__, EWL, RQ, initial lean mass, initial fat mass, diet, treatment and a diet by week interaction to capture potential differences in ^13^C tissue content with time on the experimental diets. For experiment 1, we also included the time elapsed between water removal and the RMR measurement as an initial covariate in the δ^13^C_breath_ model. We modeled total water loss as a function of total O_2_ consumed, lean and fat mass lost during respirometry, initial body mass, and treatment as fixed effects.

Mixed models were validated using the DHARMa package (https://CRAN.R-project.org/package=DHARMa). For all models, we tested the significance of the parameter estimates using the function ‘Anova’ (package car; Fox and Weisberg, 2019), specifying type-III sums of squares. When necessary, pairwise differences between groups were identified by Tukey *post hoc* tests (function ‘glht’ from package multcomp; Hothorn et al., 2008).

## RESULTS

### Experiment 1

**Fig. 2.**
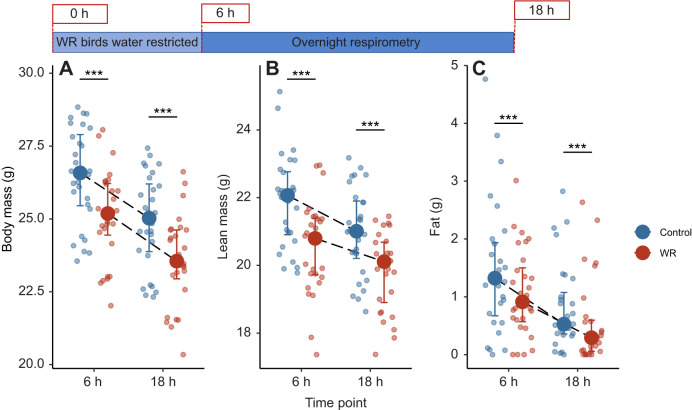
**Body composition of house sparrows before and after overnight respirometry in experiment 1.** Respirometry occurred at hours 6 and 18 of the water restriction period (top; *n*=14 birds, repeated twice per treatment). WR birds had lower overall mass (A), lean mass (B) and fat mass (C) than control birds at both time points but there were no differences in body composition change between diets or treatments. All birds lost total body mass, lean mass and fat mass during respirometry. Large circles and error bars represent the median and interquartile range; raw data points are offset within each time bin for clarity. Significant differences between treatment groups are indicated by asterisks (***P≤0.0001). See Materials and Methods for statistical details.

There were no differences in body composition between WR and control birds during respirometry, corresponding to hours 6–18 of the water restriction period (total body mass: χ^2^=0.035, *P*=0.85; lean mass: χ^2^=0.47, *P*=0.49; fat: χ^2^=0.092, *P*=0.76; Fig. 2). All birds lost mass (χ^2^=432.18, *P*<0.001) but WR birds weighed 1.34±0.42 g (5.4%) less than control birds at the end of the experiment (χ^2^=337.22, *P*<0.001; Fig. 2A). Change in total body mass was driven by loss of both lean mass (χ^2^=45.20, *P*<0.001) and fat (χ^2^=31.85, *P*<0.001; Fig. 2B,C). There were no differences in the amount of lean mass or fat lost between treatments (lean: χ^2^=0.47, *P*=0.49; fat: χ^2^=0.092, *P*=0.76), although WR birds had 1.16±0.33 g less lean mass (χ^2^=105.66, *P*<0.001) and 0.28±0.20 g less fat (χ^2^=11.22, *P*<0.001) than control birds at the end of the experiment (Fig. 2).

**Fig. 3.**
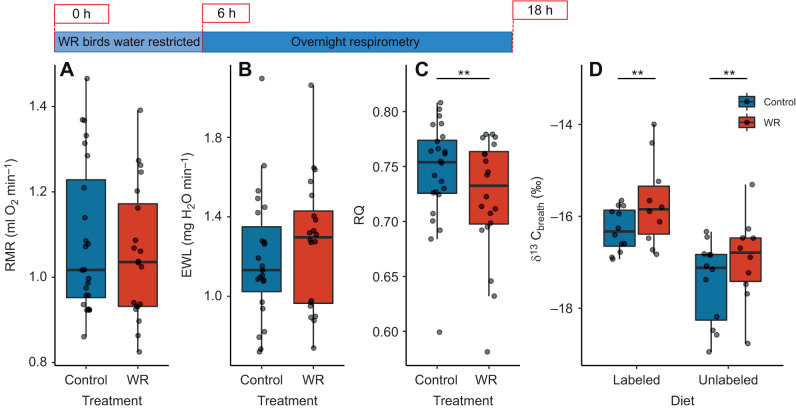
**Respirometry results for house sparrows in experiment 1.** Tukey box plots showing resting metabolic rate (RMR; A), evaporative water loss (EWL; B), respiratory quotient (RQ; C) and enrichment of ^13^C in exhaled CO_2_ (δ^13^C_breath_; D) for control birds and WR birds (*n*=14 birds, repeated twice per treatment). There were no differences in RMR or EWL between treatment groups. WR birds had lower RQ and higher δ^13^C_breath_ compared with control birds. Box plots show median, upper and lower quartiles and 1.5× interquartile range (whiskers). Raw data points are offset within each time bin for clarity. Significant differences between treatment groups are indicated by asterisks (**P≤0.01). See Materials and Methods for statistical details.

RMR did not differ between WR and control birds (χ^2^=0.0032, *P*=0.95), nor did rates of EWL (χ^2^=0.05, *P*=0.82; Fig. 3A,B). RQ decreased with RMR (χ^2^=36.82, *P*<0.001) and was higher in control birds (0.74±0.05) compared with WR birds (0.72±0.05; χ^2^=10.08, *P*=0.0015; Fig. 3C). When corrected for metabolic rate, EWL (*V̇*_H_2_O_/*V̇*_O_2__) was negatively related to initial lean mass (χ^2^=5.06, *P*=0.025). Total water loss during respirometry did not relate to lean mass loss (χ^2^=0.40, *P*=0.53) or treatment (χ^2^=1.17, *P*=0.28) but was positively related to total O_2_ consumed (χ^2^=11.22, *P*<0.001). Breath CO_2_ was enriched for ^13^C in WR birds (χ^2^=10.53, *P*=0.001; Fig. 3D). There was a significant interactive effect between week and diet on δ^13^C_breath_ (χ^2^=118.24, *P*<0.001). δ^13^C_breath_ also decreased with resting *V̇*_CO_2__ (χ^2^=13.72, *P*<0.001) and the time elapsed since the removal of water (χ^2^=9.72, *P*=0.002). All RMR measurements were obtained 8–16 h (on average, 10 h) after removing water.

### Experiment 2

**Fig. 4.**
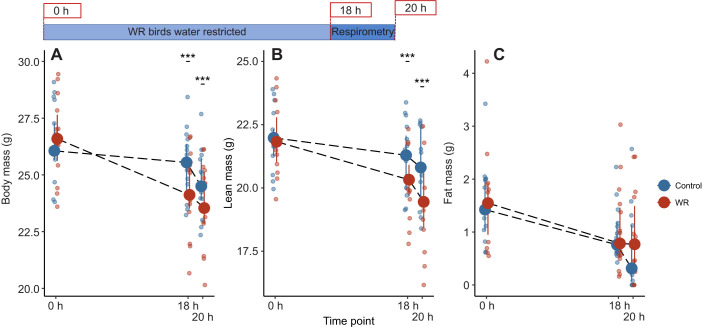
**Body composition of house sparrows before and after respirometry in experiment 2.** WR birds were water restricted starting at hour 0 while control birds were only water restricted during respirometry, which occurred between hours 18 and 20 (*n*=14 birds, repeated twice per treatment). There were no initial differences in body mass (A), lean mass (B) or fat mass (C) between treatment or diet groups. Compared with control birds, WR birds had lower body mass and lean mass at hours 18 and 20 of the water restriction period and lost more overall mass and lean mass throughout the experiment. There were no differences in fat mass between WR and control birds at any point during the experiment. All birds lost total body mass, lean mass and fat mass between hours 0, 18 and 20. Large circles and error bars represent the median and interquartile range; raw data points are offset within each time bin for clarity. Significant differences between treatment groups are indicated by asterisks (***P≤0.001). See Materials and Methods for statistical details.

We analyzed differences in body mass and lean mass between WR and control birds at hours 0, 18 and 20 separately because of significant interactions between treatment and time point. There were no initial differences in body mass (χ^2^=0.50, *P*=0.48) or lean mass (χ^2^=0.18, *P*=0.67) between treatments. Both WR and control birds lost mass between each time point (WR: χ^2^=704.17, *P*<0.001; control: χ^2^=172.03, *P*<0.001; Fig. 4A), but WR birds weighed less than control birds at hours 18 and 20 (hour 18: χ^2^=36.11, *P*<0.001; hour 20: χ^2^=50.27, *P*<0.001) due to an additional 1.35±0.65 g loss of lean mass by the end of the experiment (χ^2^=11.83, *P*<0.001; Fig. 4B). All birds lost fat during the experiment (χ^2^=54.515, *P*<0.001), but the amount of fat lost did not differ between treatments (χ^2^=0.55, *P*=0.46; Fig. 4C).

**Fig. 5.**
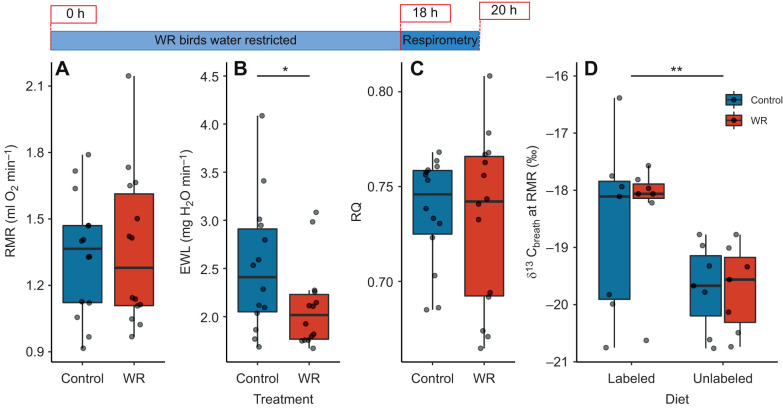
**Respirometry results for house sparrows in experiment 2.** Tukey box plots showing RMR (A), EWL (B), RQ (C) and δ^13^C_breath_ (D) of control birds and WR birds (*n*=14 birds, repeated twice per treatment). Birds fed the ^13^C-labeled leucine diet had higher δ^13^C_breath_ than birds fed the control diet; there were no differences in δ^13^C_breath_ between treatment groups. WR birds had lower EWL than control birds. Box plots show median, upper and lower quartiles and 1.5× interquartile range (whiskers). Raw data points are offset within each time bin for clarity. Significant differences between treatment groups are indicated by asterisks (*P≤0.05; **P≤0.01). See Materials and Methods for statistical details.

There were no differences in RMR or RQ between WR and control birds (RMR: χ^2^=0.26, *P*=0.61; RQ: χ^2^=0.12, *P*=0.73); however, WR birds had lower EWL than control birds (χ^2^=8.85, *P*=0.003; Fig. 5A–C). δ^13^C_breath_ did not differ between treatments (χ^2^=0.016, *P*=0.90; Fig. 5D) but was lower in birds on the unlabeled diet (χ^2^=6.75, *P*=0.009) and significantly decreased with fat mass (χ^2^=15.97, *P*<0.001), *V̇*_CO_2_ _(χ^2^=6.74, *P*=0.0094) and EWL (χ^2^=5.27, *P*=0.022).

## DISCUSSION

The protein-for-water strategy has been proposed mainly in the context of long-distance migratory flight, during which birds are constrained in their ability to stop for water and may need to rely on metabolic sources for balancing water budgets. Yet, the ability to modulate the composition of oxidative fuels to increase endogenous water production in response to dehydration may be conserved among birds, and uniquely beneficial among uricotelic animals that can efficiently excrete nitrogenous waste (McCue et al., 2017). Birds are also unique among vertebrates in their ability to fuel high-intensity endurance exercise with fat and have been shown to increase their capacity to rapidly transport and oxidize fatty acids during migration (Guglielmo, 2010). The ability to store and use large amounts of fat allows migratory birds to fuel long flights, yet flight range in ultra long-distance migrants may be ultimately constrained by protein loss (Elowe et al., 2023; Gerson et al., 2020). Despite functional consequences of lean mass loss for flight and stopover performance, protein is a crucial source of metabolites and endogenous water. Lowered body water content in migratory birds has only rarely been confirmed, such as in trans-desert migrants (Biebach, 1990), suggesting that supplemental water arising from metabolism is sufficient to balance EWL during migration except in the most extreme conditions. The importance of protein as a fuel source for migratory birds is also evidenced by seasonal changes in protein metabolism, such as in white-throated sparrows, which upregulate enzymatic pathways for protein catabolism in the migratory condition (Elowe and Gerson, 2022).

Previous studies have shown that birds preferentially catabolize proteins under conditions of water stress such as high temperature and low humidity (Gerson and Guglielmo, 2011a,b; Gerson et al., 2020, 2019); however, the question of whether birds oxidize the resulting amino acids to liberate endogenous water, or recycle amino acids in other ways, has not been directly tested. Consistent with previous studies (Gerson and Guglielmo, 2011a,b), we found that dehydrated birds had reduced lean mass (experiment 1) and lost lean mass at a greater rate (experiment 2) relative to control birds. Additionally, resting δ^13^C_breath_ was enriched in WR birds compared with control birds in birds fed ^13^C-labeled leucine (experiment 1), indicating elevated oxidation of endogenous amino acids. Increased oxidation of non-lipids, which are naturally enriched for ^13^C, in response to water restriction was also detectable in birds fed the control diet. The RQ values of WR and control birds indicate that both groups were primarily burning a mixture of fat and protein; RQ of protein metabolism and RQ of pure fat metabolism (0.74 and 0.71, respectively) are almost indistinguishable in uricotelic animals. That WR birds had slightly lower RQ values may suggest they were sparing carbohydrates, though it is unclear why. The observed increase in resting δ^13^C_breath_ in WR birds was then likely driven primarily by elevated oxidation of amino acids, and not carbohydrates.

There was no difference in δ^13^C_breath_ between WR and control birds during experiment 2, despite WR birds being water deprived 3 times longer before respirometry was conducted. Additionally, resting δ^13^C_breath_ declined over time during experiment 1 and was higher on average relative to experiment 2, suggesting that birds burned proportionally more fat later in the fasting period regardless of hydration status. During prolonged fasting, initial high rates of protein catabolism (phase I) decline to a minimum steady state (phase II) while contributions of fat to energy expenditure are maximized (Cherel et al., 1988). This strategy reserves protein until fat stores drop below a set threshold (initiating phase III), at which point lean mass catabolism dramatically increases (Jenni et al., 2000). The composition of fuels used in a fasting state has been found previously to depend on the initial body condition of a bird, as does the length of phase II of starvation (Jenni-Eiermann and Jenni, 2012). Similarly, we found that δ^13^C_breath_ during hours 18–20 of the water restriction period in experiment 2 was related to initial fat mass, indicating that the proportion of fat in the fuel mixture was dependent on the size of fat stores.

The QMR data indicate that WR birds did catabolize protein in the period before respirometry was conducted during experiment 2 (hours 0–18), as they lost lean mass at a greater rate than control birds, aligning temporally with our observation of enriched δ^13^C_breath_ earlier in the water restriction period in experiment 1. This evidence of dynamic protein use, and elevated rates of protein catabolism early in periods of water stress, is supported by observations of non-linear changes in lean mass catabolism in fasted yellow-rumped and blackpoll warblers; these birds, both resting and flying, exhibited high initial rates of lean mass loss that declined exponentially over time (Elowe et al., 2023). Birds may minimize rates of protein catabolism to spare protein during prolonged periods of dehydration by using alternative strategies to reduce rates of water loss. We found that relative to control birds, WR birds had reduced EWL during experiment 2 but showed no difference in δ^13^C_breath,_ while WR birds in experiment 1 had enriched δ^13^C_breath_ but similar rates of EWL. It is possible that by hour 18 of water restriction, WR birds had engaged physiological or behavioral strategies to minimize water loss, reducing the need to elevate metabolic water production and allowing them to spare protein in favor of fat catabolism. Depending on the conditions causing dehydration, birds can reduce water loss by retaining feces in the digestive tract longer to increase water reabsorption or by using adaptive hyperthermy to reduce water lost through evaporative cooling, among other strategies (Arad et al., 1987; Brischoux et al., 2020; Gerson et al., 2019; Moldenhauer and Taylor, 1973; Goldstein and Braun, 1988). It should be noted, however, that absolute rates of EWL were higher for both control and WR birds during experiment 2, likely because the respirometry trial was conducted during the day when RMR is greater or because the duration was too short for birds to reach RMR.

When rates of water loss are low or metabolic rates are high, endogenous water production resulting from the catabolism of fat may be sufficient for keeping water budgets balanced. Species that are adapted to arid environments may have evolved other physiological strategies for maintaining water balance in response to dehydration stress. Zebra finches (*Taeniopygia guttata*) fasting without water, for example, were able to maintain water balance by elevating fat catabolism alone (Rutkowska et al., 2016). Water-restricted house sparrows increased rates of lean mass catabolism at rest but not during a metabolic challenge, indicating that a shift in fuel mixture to include proportionally more protein was not necessary under conditions of elevated metabolic rate in the cold (Gerson and Guglielmo, 2011a). In the present study, we found that δ^13^C_breath_ decreased with metabolic rate in both experiments, indicating that fuel mixtures with proportionally more fat produced enough metabolic water at high metabolic rates to offset water losses. This result also suggests that the generation of citric acid cycle intermediates is likely not a strong driver of protein catabolism (Jenni and Jenni-Eiermann, 1998). If it was, δ^13^C_breath_ would increase proportionally with metabolic rate.

### Conclusions

The results of these experiments provide novel evidence that at least one function of lean mass catabolism in water-stressed birds is to increase metabolic water synthesis via endogenous amino acid oxidation. We cannot discount the possibility that proteins are also catabolized to modulate cellular osmolality in response to dehydration. Nor do our results discount other non-mutually exclusive hypotheses regarding the function of protein catabolism during flight in birds, such as generating metabolites for gluconeogenesis. We did find clear evidence, however, that birds increase the oxidation of endogenous amino acids in response to water stress, informing our understanding of how animals cope with water stress and possible limits to such tolerance. There are still many outstanding questions regarding the drivers and functions of protein use in birds and other animals that warrant further research, such as the temporal dynamics of protein oxidation during dehydration and the impacts of lean mass catabolism on other physiological systems. These questions have broad implications regarding migration behavior, thermoregulation and metabolism.

## ECR Spotlight

This article has an associated ECR Spotlight interview with Elizabeth Rogers.
